# Asymptomatic Meibomian Gland Dysfunction and Cardiovascular Disease Risk Factors in a Middle-Aged Population in Taiwan - A Cross-sectional Analysis

**DOI:** 10.1038/s41598-017-05368-z

**Published:** 2017-07-10

**Authors:** Alexander Chen, Hung-Ta Chen, Hung-Chi Chen, Yi-Tsun Chen, Yih-Hsiou Hwang, Chi-Chin Sun, Ching-Hsi Hsiao, David Hui-Kang Ma, Wei-Chi Wu, Chi-Chun Lai

**Affiliations:** 1 Department of Ophthalmology, Chang Gung Memorial Hospital, Linkou, Taiwan; 2 Department of Medicine, Chang Gung University College of Medicine, Taoyuan, Taiwan; 3 Department of Internal Medicine, Taipei City Hospital- Heping Branch, Taipei, Taiwan; 4 Center for Tissue Engineering, Chang Gung Memorial Hospital, Linkou, Taiwan; 5 Department of Family Medicine, Taipei Veterans General Hospital, Taipei, Taiwan; 6 Department of Ophthalmology, Chang Gung Memorial Hospital, Keelung, Taiwan

## Abstract

Managing cardiovascular disease (CVD) risk factors is the key to prevent CVD. This study aimed to prevent CVD by introducing asymptomatic meibomian gland dysfunction (MGD), a condition associated with various CVD risk factors, as an early indicator for CVD in middle-aged population. Participants with and without asymptomatic MGD underwent standardized questionnaires, physical examinations, and laboratory investigations. One ophthalmologist completed the identification and grading of MGD by using slit-lamp biomicroscopy examination on the eyelid margins, meibomian gland orifices, and meibomian gland secretions. Standardized techniques were used to measure the CVD risk factor parameters. After adjusted for age and gender, CVD risk factors including elevated uric acid (P = 0.01), total cholesterol (Total-C, P < 0.001), low-density lipoprotein cholesterol (LDL-C, P < 0.001), fasting triglyceride (Fasting TG, P < 0.001), decreased high-density lipoprotein cholesterol (HDL-C, P = 0.04), and presence of hepatic steatosis (P = 0.008) were significantly associated with asymptomatic MGD. Stepwise logistic regression analysis revealed that LDL-C (OR: 1.03, 95% CI: 1.02–1.04) and Fasting TG (OR: 1.01; 95% CI: 1.00–1.01) levels were risk factors for having asymptomatic MGD (P < 0.001). Together, the results suggest that asymptomatic MGD may serve as an indicator for CVD.

## Introduction

Meibomian glands, or a row of sebaceous, holocrine glands that lie along the lid margin, are responsible for the lipid portion of the tear film^1^ through the secretion of meibum, which not only facilitates spread of the tear by retarding its evaporation, but also acts as a barrier against contamination^2^. Frequently encountered in clinical practice^1^, meibomian gland dysfunction (MGD) is characterized by outflow obstruction, increased meibum viscosity, duct dilatation, hyposecretion, and even acinar atrophy, which causes patient great distress. Several systemic factors such as aging^3^, androgen deficiency^4, 5^, anti-androgen use^5^, atopy^6^, menopause^5^, Parkinson’s disease^7^, rosacea^8^, Sjogren’s syndrome^9^, and Stevens-Johnson syndrome^10^ have been reported to be correlated with MGD. Given the lipid component of meibomian gland’s secretion, it is plausible to deduce that disorders regarding production or metabolism of lipid may also lead to MGD. Recently, numerous studies have found correlation between symptomatic MGD and dyslipidemia^11–14^. It remains unexplored whether this correlation still holds true in the population with asymptomatic MGD.

The importance of dyslipidemia lies in the fact that it is one of the major risk factors in cardiovascular disease (CVD)^15^. However, dyslipidemia is the only factor that has been discussed in conjunction with MGD, while other CVD risk factors such as obesity, diabetes mellitus, hypertension, metabolic syndrome, high-sensitivity C-reactive protein (hs-CRP), age, sex, uric acid level, and the presence of hepatic steatosis have not. Although it is well known that the above CVD risk factors^16–19^ as well as MGD^11–14^ are related to lipid and glucose metabolism, it remains unknown whether MGD is correlated with CVD risk factors.

One can never overemphasize the importance of early detection of CVD, which is one of the most common cause of death and also greatly impacts the lives of middle-aged population and their families. Having early prevention of CVD in mind, and that asymptomatic MGD is more common than symptomatic MGD^20^, current study aimed to identify the correlation between asymptomatic MGD and CVD risk factors in a middle-aged population using the screening profiles obtained during the participants’ voluntary physical examinations and laboratory investigations.

## Results

The prevalence of asymptomatic MGD among middle-aged, community-based participants was 89/1329 (6.7%). The participant’s baseline characteristics and metabolic status with and without asymptomatic MGD are listed in Table 1. Most participants with asymptomatic MGD were men (86.5%). Participants with asymptomatic MGD had higher mean serum concentrations of Total-C (*P* < 0.001), LDL-C (*P* < 0.001), Fasting TG (*P* < 0.001), uric acid (*P* = 0.02), and higher probability of hepatic steatosis (*P* = 0.002) than those without. Conversely, participants with asymptomatic MGD had lower mean serum concentrations of HDL-C (*P* = 0.030) than those without. There were no significant differences in terms of WC, BMI, SBP, DBP, AC, PC, hs-CRP, age, sex, and the presence of metabolic syndrome between these two groups with or without asymptomatic MGD.

**Table 1:** Baseline Characteristics and Metabolic Status of Participants with and without Asymptomatic Meibomian Gland Dysfunction.

	Without MGD	With MGD	*P* value
Participants (n)	199	89	
Age (yr)	48.7 (12.6)	49.0 (10.4)	0.82
Male [n(%)]	159 (79.9)	77 (86.5)	0.18
WC (cm)	84.1 (9.9)	86.0 (9.5)	0.12
BMI (kg/m^2^)	23.9 (3.4)	24.4 (3.2)	0.24
Systolic BP (mmHg)	131.1 (17.6)	134.3 (19.8)	0.17
Diastolic BP (mmHg)	80.4 (11.1)	82.4 (12.6)	0.18
Fasting glucose (mg/dL)	95.0 (22.9)	98.3 (30.2)	0.31
Postprandial glucose (mg/dL)	104.5 (47.5)	108.6 (50.5)	0.50
Uric acid (mg/dL)	6.0 (1.6)	6.5 (1.7)	**0**.**02**
Total-C (mg/dL)	187.6 (34.6)	213.4 (34.4)	<**0**.**001**
LDL-C (mg/dL)	107.9 (29.7)	134.7 (29.5)	<**0**.**001**
Fasting TG (mg/dL)	129.7 (74.2)	188.4 (109.3)	<**0**.**001**
HDL-C (mg/dL)	51.3 (13.2)	47.7 (11.9)	**0**.**030**
hs-CRP (mg/dL)	1.9 (2.6)	1.7 (2.4)	0.57
Presence of hepatic steatosis [n (%)]*	103 (55)	61 (74)	**0**.**002**
Presence of metabolic syndrome [n (%)]	56 (28)	34 (38)	0.09

BMI = body mass index; BP = blood pressure; HDL-C = high-density lipoprotein cholesterol; hs-CRP = high-sensitivity C-reactive protein; LDL-C = low-density lipoprotein cholesterol; MGD = meibomian gland dysfunction; TG = triglyceride; Total-C = total cholesterol; WC = waist circumference.

*Only 271 out of 288 (total) participants have undergone abdominal ultrasonography for hepatic steatosis.

Values are expressed as average (standard deviation).

Correlations significant at *P* < 0.05 are bolded.

### Correlations between Asymptomatic Meibomian Gland Dysfunction and Cardiovascular Disease Risk Factors

To assess the correlations between asymptomatic MGD and CVD risk factors, we divided continuous variables into three tertiles (Table 2). After adjusted for age and gender, participants having elevated uric acid (*P* = 0.01), Total-C (*P* < 0.001), LDL-C (*P* < 0.001), Fasting TG (*P* < 0.001), decreased HDL-C (*P* < 0.04), as well as having hepatic steatosis (*P* < 0.008) were significantly associated with asymptomatic MGD. No significant correlations were detected between asymptomatic MGD and parameters such as WC, BMI, SBP, DBP, AC, PC, and hs-CRP.

**Table 2:** Correlations between Asymptomatic Meibomian Gland Dysfunction and Cardiovascular Disease Risk Factors.

		Crude rate of MGD, n (%)	Adjusted Odds ratio (95% CI)^†^	*P v*alue for trend
WC (cm)	<80	22 (26)	1	
	80–88	35 (34)	1.47 (0.22–2.79)	
	≧89	32 (33)	1.38 (0.70–2.71)	0.41
BMI (kg/m^2^)	<22.7	23 (24)	1	
	22.7–25.0	30 (34)	1.46 (0.77–2.76)	
	≧25.1	34 (34)	1.52 (0.81–2.85)	0.24
Systolic BP (mmHg)	<124	29 (30)	1	
	124–138	23 (26)	0.88 (0.45–1.71)	
	≧139	37 (37)	1.41 (0.74–2.69)	0.22
Diastolic BP (mmHg)	<76	27 (26)	1	
	76–84	28 (33)	1.08 (0.57–2.05)	
	≧85	34 (34)	1.22 (0.64–2.30)	0.48
Fasting Glucose (mg/dL)	<87	24 (26)	1	
	87–93	28 (30)	1.24 (0.64–2.37)	
	≧94	37 (36)	1.67 (0.86–3.22)	0.27
Postprandial Glucose (mg/dL)	<86	24 (25)	1	
	86–100	31 (33)	1.77 (0.92–3.41)	
	≧101	34 (35)	1.86 (0.94–3.68)	0.26
Uric Acid (mg/dL)	<5.3	20 (21)	1	
	5.3–6.6	29 (31)	1.62 (0.83–3.17)	
	≧6.7	40 (40)	**2**.**40** (**1**.**23**–**4**.**70**)	**0**.**01**
Total-C (mg/dL)	<178	17 (19)	1	
	178–209	24 (24)	1.47 (0.73–2.97)	
	≧210	48 (49)	**4**.**24** (**2**.**19**–**8**.**22**)	<**0**.**001**
LDL-C (mg/dL)	<99	10 (11)	1	
	99–126	27 (28)	**3**.**06** (**1**.**38**–**6**.**76**)	
	≧127	52 (53)	**9**.**05** (**4**.**19**–**19**.**55**)	<**0**.**001**
Fasting TG (mg/dL)	<96	18 (19)	1	
	96–156	23 (24)	1.36 (0.68–2.74)	
	≧157	48 (48)	**3**.**90** (**2**.**02**–**7**.**54**)	<**0**.**001**
HDL-C (mg/dL)	≧55	19 (21)	1	
	44–54	37 (36)	**2**.**02** (**1**.**04**–**3**.**90**)	
	<44	33 (35)	1.91 (0.97–3.77)	**0**.**04**
hs-CRP (mg/dL)	<0.75	30 (31)	1	
	0.75–1.65	28 (30)	0.98 (0.52–1.82)	
	≧1.70	31 (31)	1.04 (0.56–1.91)	0.98
Presence of Hepatic Steatosis [n (%)]*	No	21 (20)	1	
	Mild	37 (39)	**2**.**15** (**1**.**19**–**3**.**88**)	
	Moderate-to-severe	24 (35)	1.77 (0.92–3.44)	**0**.**008**

BMI = body mass index; BP = blood pressure; HDL-C = high-density lipoprotein cholesterol; hs-CRP = high-sensitivity C-reactive protein; LDL-C = low-density lipoprotein cholesterol; MGD = meibomian gland dysfunction; MS = metabolic syndrome; TG = triglyceride; Total-C = total cholesterol; WC = waist circumference.

*Only 271 out of 288 (total) participants have undergone abdominal ultrasonography for hepatic steatosis.

^†^Adjusted for age and gender.

Correlations significant at *P* < 0.05 are bolded.

### Risk Factors for Asymptomatic Meibomian Gland Dysfunction by using Multivariate Logistic Regression

The risk factors for asymptomatic MGD via multivariate logistic regression analysis were documented in Table 3. Higher LDL-C was a significant and independent risk factor for asymptomatic MGD (OR: 1.03; 95% CI: 1.02–1.04). Higher Fasting TG was also a significant but milder risk (OR: 1.01; 95% CI: 1.00–1.01) for asymptomatic MGD. Both results were consistent with the above findings.

**Table 3:** Risk Factors for Asymptomatic Meibomian Gland Dysfunction by using Multivariate Logistic Regression.

Risk factors	Adjusted Odds Ratio (95% CI)*	*P* value
LDL-C	1.03 (1.02–1.04)	<**0**.**001**
Fasting TG	1.01 (1.00–1.01)	<**0**.**001**

CI = confidence interval; MGD = meibomian gland dysfunction.

Variables enrolled in this analysis include sex, age, waist circumference, body mass index, systolic blood pressure, diastolic blood pressure, fasting glucose, postprandial glucose, fasting triglyceride (Fasting TG), high-density lipoprotein cholesterol, low-density lipoprotein cholesterol (LDL-C), uric acid, high-sensitivity C-reactive protein, presence of metabolic syndrome, and presence of hepatic steatosis.

*Adjusted for age and gender.

Correlations significant at *P* < 0.05 are bolded.

### Associations between the Severity of Asymptomatic Meibomian Gland Dysfunction and Risk of Having Dyslipidemia

To further evaluate the correlations between asymptomatic MGD and CVD risk factors, participants were categorized into three groups via findings in the slit lamp: no MGD, mild MGD, and moderate-to-severe MGD. We then calculated the odds ratio of dyslipidemia in terms of four indicators including Total-C, LDL-C, HDL-C and Fasting TG (Table 4). After adjusted for age and gender, analysis showed significant association between asymptomatic MGD and dyslipidemia except HDL-C, including Total-C ≧ 240 mg/dL, LDL-C ≧ 160 mg/dL, and Fasting TG ≧ 150 mg/dL. Also, the participants with severe asymptomatic MGD also had significantly higher risk of having Fasting TG ≧ 160 mg/dL than those who had asymptomatic MGD (OR: 2.70 versus 5.46).

**Table 4:** Associations between the Severity of Asymptomatic Meibomian Gland Dysfunction and Risk of Having Dyslipidemia.

	Adjusted Odds Ratio (95% CI)* for Having Dyslipidemia			
	Total-C ≧ 240 mg/dL	LDL-C ≧ 160 mg/dL	Fasting TG ≧ 150 mg/dL	HDL-C < 45 mg/dL
Without Asymptomatic MGD	1	1	1	1
Mild	2.08	2.68	**2**.**70**	1.16
Asymptomatic MGD	(0.79–5.52)	(0.91–7.94)	(**1**.**41**–**5**.**16**)	(0.61–2.22)
Moderate-to-severe	**7**.**91**	**5**.**82**	**5**.**46**	1.63
Asymptomatic MGD	(**3**.**40**–**18**.**37**)	(**2**.**16**–**15**.**67**)	(**2**.**53**–**11**.**79**)	(0.79–3.36)

CI = Confidence Interval; Fasting TG = fasting triglyceride; HDL-C = high-density lipoprotein cholesterol; LDL-C = low-density lipoprotein cholesterol; MGD = meibomian gland dysfunction; Total-C = total cholesterol.

*Adjusted for age and gender.

Correlations significant at *P* < 0.05 are bolded.

## Discussion

In order to establish the relationship between asymptomatic MGD and CVD, it is imperative to first identify the potential CVD risk factors for asymptomatic MGD. In this study, we found that dyslipidemia, uric acid, and hepatic steatosis were significantly associated with asymptomatic MGD. Furthermore, increased level of Total-C, LDL-C, Fasting TG, and decreased level of HDL-C were positively correlated with the severity of asymptomatic MGD.

The design of this study was built on the notion that meibomian gland, a sebaceous gland secreting the lipid layer of the tear film, is related to serum lipid. In fact, in 2008, Butovich *et al*. confirmed that the major component of human meibum is cholesteryl ester, an inactive form of cholesterol for the efficiency of transportation^21^. Consequently, this implies that the abnormality of serum lipid and glucose metabolism results in diseases such as MGD, CVD, as well as other risk factors of CVD. Therefore, it is crucial to discuss the association between MGD and dyslipidemia. Although some studies in the past have suggested that meibomian gland is capable of synthesizing lipid de novo^22, 23^, more recent studies, although differing in various degrees, have found correlations between meibomian lipids and serum lipid profile levels (Table 5). In 2010, Dao *et al*. studied the association between dyslipidemia and moderate to severe MGD and found a higher prevalence of elevated Total-C in patients with MGD than those without. In addition, since the incidence of decreased HDL-C levels was actually lower in patients with MGD, they concluded that it is mainly the elevated HDL-C levels that contributed to the increased Total-C^13^. In 2013, Bukhari analyzed the correlation between the severity of MGD and the severity of dyslipidemia and found no correlation between the two, but the severity of MGD is correlated with the increased prevalence of high Fasting TG and LDL-C levels^12^. Pinna *et al*., in 2013, investigated the correlation between MGD and hypercholesterolemia in young and middle-aged participants and their results, like Braich *et al*.’s study in 2015, showed that participants with MGD have higher mean levels of Total-C, LDL-C, Fasting TG, and HDL-C than those without. Nonetheless, the results differed in that Pinna *et al*. found MGD to be associated with higher serum concentrations of Total-C, LDL-C, and HDL-C as compared to Braich *et al*.’s higher serum concentrations of Total-C, LDL-C, and Fasting TG^11, 14^. Our results were mostly in agreement with the previous findings in that participants with asymptomatic MGD had higher mean levels of Total-C, LDL-C, and Fasting TG than those without. However, lower mean level of HDL-C was observed in our study for participants with asymptomatic MGD than those without. In addition, after adjusted for age and gender, elevated Total-C, LDL-C, Fasting TG, and decreased HDL-C were significantly associated with asymptomatic MGD. Through multivariate logistic regression analysis, higher LDL-C and Fasting TG were independent risk factors for asymptomatic MGD (Table 3). Concisely, the most surprising findings were the lower mean level of HDL-C in participants with asymptomatic MGD, as well as the association between decreased HDL-C and asymptomatic MGD, which contradicted with the previous studies. We think that dyslipidemia may be the cause of MGD, but whether it is symptomatic or asymptomatic lies with the higher and lower serum level of HDL-C, respectively. Still, further studies are needed to support the observation. Despite the above discrepancies between our study and the previous ones, the lipid profile in our participants with asymptomatic MGD, specifically the lower mean HDL-C level, seemed to coincide with the current belief of what is known as susceptible to CVD^18^. Importantly, this should be the first study to have identified a correlation between the severity of asymptomatic MGD and the risk of dyslipidemia. Namely, the severity of asymptomatic MGD is positively correlated with increased risks of Total-C, LDL-C, and Fasting TG (Table 4). Collectively, based on our association analysis between dyslipidemia and asymptomatic MGD, as well as the correlation analysis between the severity of asymptomatic MGD and the risk of dyslipidemia, we speculate that asymptomatic MGD may be associated with CVD.

**Table 5:** Comparison of Key Findings between Current and Previous Studies.

	Dao *et al*.^13^	Bukhari *et al*.^12^	Pinna *et al*.^14^	Braich *et al*.^11^	Chen *et al*. 2017
Total number of participants	46	236	123	224	288
Age of all participants (mean)	27–82 (52)	15–78 (49.4)	18–54	20–75	30–60 (48.9)
Age of patients with MGD (mean)	N/A	N/A	18–54 (37.7)	20–72 (46.7)	30–60 (49)
Age of controls (mean)	N/A	N/A	18–54 (35.9)	19–75 (45.9)	30–60 (48.7)
Mean level differences between participants with and without MGD					
Male	NSD	NSD	NSD	NSD	NSD
Female	NSD	NSD	NSD	NSD	NSD
Total-C	HI	NSD	Higher	Higher	Higher
LDL-C	NSD	HI	Higher	Higher	Higher
Fasting-TG	LI	HI	Higher	Higher	Higher
HDL-C	HI	NSD	Higher	Higher	Lower
BMI (mean)	N/A	N/A	NSD	NSD	NSD
Glucose	N/A	N/A	NSD	NSD	NSD
Creatinine	N/A	N/A	NSD	NSD	N/A
Association with MGD:					
Adjusted odds ratio (95% CI)					
Higher Total-C	N/A	N/A	1.07 (1.04–1.09)	14.3 (8.2–20.7)	4.24 (2.19–8.22)
Higher LDL-C	N/A	N/A	1.07 (1.04–1.09)	9.1 (6.6–13.2)	9.05 (4.19–19.55)
Higher Fasting-TG	N/A	N/A	NSD (1.9–4.4)	3.2 (2.02–7.54)	3.9
Higher HDL-C	N/A	N/A	1.11 (1.06–1.17)	NSD	2.02 (Low HDL-C) (1.04–3.90)

CI = confidence interval; HDL-C = high-density lipoprotein cholesterol; HI = higher incidence; LDL-C = low-density lipoprotein cholesterol; LI = lower incidence; MGD = meibomian gland dysfunction; N/A = not available; NSD = no significant differences; TG = triglyceride; Total-C = total cholesterol.

An interesting finding in our study was the association between the serum uric acid level and asymptomatic MGD. We not only found a higher serum uric acid level in the asymptomatic MGD group in comparison to that of the control, after adjusted for age and gender, we also found positive association between uric acid and asymptomatic MGD. Previously, it was detected that the mean level of uric acid for participants with (6.0 mg/dL) and without MGD (6.5 mg/dL) are both lower than the normal mean of 6.7 mg/dL for Taiwanese population according to Gosling *et al*.’s review^24^. One way to interpret the observed lower-than-average uric level in both groups was that our participants were collected from one single medical center and happened to be lower than the entire Taiwanese population. A second possible explanation was that Gosling *et al*. only collected male data to avoid the confounding hormone effects on uric acid level in pre-menopausal women^24^, which resulted in such bias. Nonetheless, the relationship between uric acid and asymptomatic MGD needs to be elucidated. Several studies have shown that serum uric acid level is associated with dyslipidemia, obesity, insulin resistance, hypertension^25, 26^, and metabolic syndrome^16^. Zhu *et al*.’s experimental study on mice showed that serum uric acid increases oxidative stress and reactive oxygen species, which leads to insulin resistance and abnormal glucose metabolism^27^. Knowing that glucose and lipid are molecularly interchangeable, perhaps this explains the reason that the above risk factors as well as asymptomatic MGD, are associated with CVD.

Our discovery of the association between the presence of hepatic steatosis and asymptomatic MGD was also an intriguing finding. A systematic review and meta-analysis by Gong *et al*. disclosed that hyperuricemia is associated with increased risk for nonalcoholic hepatic steatosis^28^. Studies have also shown that nonalcoholic hepatic steatosis is highly associated with metabolic syndrome, diabetes^29^, dyslipidemia, and cardiovascular disease^30, 31^. The specific mechanisms of fatty liver formation have not been well delineated and are currently believed to be multifactorial. Nonetheless, Rinella *et al*.’s systematic review proposed that the alteration of lipid and glucose metabolism generated by visceral adipose tissues is a major cause of fat buildup, which consequently causes organ inflammations and injuries^32^. This explanation concurs with our observation and further justifies that asymptomatic MGD is also the result of alteration in lipid and glucose metabolism, and therefore is associated with CVD risk factors.

In spite of the above findings that support the association of asymptomatic MGD and CVD, we did not observe any associations among asymptomatic MGD and other CVD risk factors that are supposedly interrelated to dyslipidemia: these include the WC, BMI, SBP, DP, fasting glucose, PC glucose, and metabolic syndrome. Obesity is the consequence of fat accumulation, and is inevitably interrelated to dyslipidemia. Common assessments for obesity include BMI and WC. Lately, studies have found no differences in BMI between groups with and without asymptomatic MGD^12, 14^. In addition to BMI, we have included WC for obesity indicator to reinforce the evaluation. Unfortunately, no significant differences were observed among MGD, BMI and WC in groups with and without asymptomatic MGD. Obesity and hepatic steatosis are both the result of lipid accumulation, but only hepatic steatosis was found to be correlated with asymptomatic MGD. A possible explanation is that visceral adiposity specifically^33^, rather than fat accumulation in general^34^, is the major contributor to the complications of obesity. As for blood pressure, it is well known that most causes of hypertension cannot be determined. Hence, even though there are many factors that may contribute to hypertension, they all play a certain but limited role. Similar to our results, those published by Viso *et al*. also failed to demonstrate any significant difference in blood pressure between patients with and without asymptomatic MGD^20^. With regard to hyperglycemia, Viso *et al*. found that asymptomatic MGD, but not symptomatic MGD, is associated with diabetes, which is unexpected and difficult to explain. More recent studies reinforced the idea by looking at the serum glucose level but found no significant differences between groups with and without MGD^12, 14^. In our study, fasting glucose and PC glucose were, for the first time, added to the comparison, but no significant differences were found between groups with and without asymptomatic MGD. Finally, it makes sense to include metabolic syndrome into this study, for metabolic syndrome encompasses dyslipidemia, visceral obesity, hypertension, and hyperglycemia^19^ and is known to greatly increase the risk of CVD^35^. To our knowledge, this is the first study to illustrate the relation between metabolic syndrome and asymptomatic MGD. Unfortunately, no association were found in this study. Taken together, in order to better evaluate the association among the above CVD risk factors and asymptomatic MGD, visceral rather than general obesity should be focused; on the other hand, too many disrupting mechanisms are involved in hypertension and hyperglycemia, which lead to the lack of association between the presence of metabolic syndrome and asymptomatic MGD. Accordingly, further studies are needed to unravel the missing links.

We were also unable to identify any association among asymptomatic MGD, hs-CRP, age, and gender. This should be the first and only report on the relation of hs-CRP and asymptomatic MGD. It is well known that hs-CRP is a non-specific inflammatory biomarker. Other studies also demonstrated that CRP can be found in atherosclerotic plaques that are responsible for acute coronary syndromes^36^, implicating that the elevated hs-CRP observed may be mainly attributed to atherosclerotic involvements and may not necessarily have any relation with asymptomatic MGD. The lack of association in hs-CRP between groups with and without asymptomatic MGD, therefore, probably depended on the underlying atherosclerotic involvement. As for the lack of difference in age between groups with and without MGD, our study was preconditioned so that the participants enrolled were between the age of 30 and 60 in order to focus on the middle-aged population, which inevitability reduced the variation. The strength of this preconditioning was that the differences in gender between groups with and without asymptomatic MGD could be more evidently identified. In a recent study by Braich *et al*.^11^, the odds of having MGD is moderately increased in the population of elderly (age > 65) male. Our study, which focused on the middle-aged population, found no such difference in terms of sex, suggesting that the etiology of asymptomatic MGD involving gender differences is of late-onset, but the exact mechanism is still uncertain.

There were some limitations in our study. Firstly, the ophthalmic examinations were completed by a single ophthalmologist, which minimized the interobserver error but at the same time reduced the reproducibility. Secondly, all our participants were collected from a single medical center, resulting in selection bias. Thirdly, the majority of participants with (86.5%) and without asymptomatic MGD (79.9%) were comprised of male, which made the differences between gender difficult to distinguish. Lastly, our retrospective cross-sectional study failed to demonstrate the temporal sequence between asymptomatic MGD and the CVD risk factors.

In summary, in the middle-aged Taiwanese population, our study suggests that the association between asymptomatic MGD and CVD is dependent on elevated Total-C, LDL-C, Fasting TG, decreased HDL-C, increased uric acid, and the presence of hepatic steatosis. Since early diagnosis of CVD can increase the survival rate drastically, we recommend that MGD screening should be used for such purpose. Further larger prospective studies on the association between asymptomatic MGD and CVD are also warranted.

## Methods

### Participant Recruitment

This study was approved by the Institutional Review Board of Chang Gung Memorial Hospital, Linkou, Taiwan (Registration Number: 201600086B0) and adhered to the tenets of the Declaration of Helsinki.

A retrospective single-center cross-sectional study of eyes with asymptomatic MGD from middle-aged participants was conducted. The clinical records of 1,979 participants registered for the Health Care Program at the Chang Gung Memorial Hospital during an interval of four years were reviewed. Participants with age between 30 and 60 years old were enrolled (n = 1,329). The exclusion criteria included those with thyroid dysfunction, alcoholism, dyslipidemia under lipid-lowering therapy, positive serology for hepatitis B or hepatitis C virus, and ophthalmic abnormalities such as cataract, glaucoma, uveitis, previous ocular surgery, or symptomatic MGD. A group of participants with asymptomatic MGD (n = 89) who met the criteria was eligible for this study. Likewise, another group of age-matched participants (n = 199) who met the identical criteria, except that MGD was absent in all eyes, was used for the control group.

### History Taking and Physical Biometrics

Baseline characteristics, including history takings and physical biometrics, were carried out by the medical personnel in the Center of Health Promotion at Chang Gung Memorial Hospital. Demographic data as well as medical and surgical histories were recorded during history takings. Baseline characteristics such as systolic blood pressure (SBP), diastolic blood pressure (DBP), waist circumference (WC), presence of metabolic syndrome, body weight, and height (calculated into BMI, kg/m^2^) were obtained. Asymptomatic MGD was determined through modified questionnaire^37^ by excluding the six symptoms: the sensation of dryness, grittiness, burning, redness, lash crusting, and eyelids getting stuck.

### Slit-lamp Biomicroscopy

The ophthalmic examinations of all participants were completed by a single ophthalmologist (Chen HC) using SL-3E slit-lamp biomicroscopy (Topcon, Paramus, New Jersey). The eyelid margins, meibomian gland orifices, and meibomian gland secretions were carefully examined for signs of MGD and these were classified according to modified previous criteria^38^. MGD was graded on a scale of 0 (no obstruction) to 4 (complete obstruction). Meibum quality was graded on a scale of 0 (clear) to 4 (toothpaste-like). Participants graded 2 or lower on either of these scales in either eyes were diagnosed with mild MGD, while others moderate-to-severe.

### Laboratory Investigation

Serum lipid profiles including total cholesterol (Total-C), low-density lipoprotein cholesterol (LDL-C), high-density lipoprotein cholesterol (HDL-C), and fasting triglyceride (Fasting TG) were obtained in all participants. Fasting glucose (AC), 2-hour postprandial glucose (PC), uric acid, and hs-CRP were also checked in sera from all participants. All biochemical data were measured by the Department of Clinical Laboratory at Chang Gung Memorial Hospital. The lipid profiles were determined by enzymatic colorimetric method (Total-C and Fasting TG) and accelerator selective detergent method (LDL-C and HDL-C) using 7600-210 Clinical Analyzers (Hitachi, Tokyo, Japan). Dyslipidemia was defined as Total-C ≧ 240 mg/dL, LDL-C ≧ 160 mg/dL, and HDL-C < 40 mg/dL^18^.

### Abdominal Ultrasonography

Abdominal ultrasonography was performed and interpreted by the gastroenterologists using HDI-5000 SonoCT (Philips Co., Bothell, Washington) in all participants. Hepatic steatosis, also known as fatty liver disease, was diagnosed by characteristics of echoic patterns according to conventional criteria^39^. Hepatic steatosis was graded into mild and moderate-to-severe groups according to the ultrasonic findings.

### Statistical Analysis

Unless otherwise specified, data were presented in the forms of means ± standard deviation or frequencies. A *P* value < 0.05 was considered as statistically significant. Odds ratio with 95% confidence interval was used to compare the possibility of asymptomatic MGD. All data were analyzed using SPSS software version 13.0 (SPSS Inc. Chicago, Illinois).

To compare the differences of basic characteristics and cardiovascular risk factors between participants with and without asymptomatic MGD, Student’s *t* test and χ^2^ test were used to compare the means of continuous variables and gender differences, respectively.

To assess the correlations between asymptomatic MGD and potential CVD risk factors, continuous variables were divided into tertiles, and standardized relative risks of asymptomatic MGD were then calculated by comparing the rates of asymptomatic MGD in each group relative to that of the lowest group. Trends of the rate of asymptomatic MGD among these three groups were assessed by χ^2^ test. Stepwise multiple logistic regression analysis was performed to identify the independent risk factors associated with asymptomatic MGD.

To further evaluate the associations between intensity of asymptomatic MGD and dyslipidemia, age- and gender-adjusted relative risks were calculated for the presence of mild or moderate-to-severe MGD related to the absence of MGD.

### Data Availability

The datasets for the analysis of the current study are readily available from the corresponding author on reasonable request.
